# HseSUMO: Sumoylation site prediction using half-sphere exposures of amino acids residues

**DOI:** 10.1186/s12864-018-5206-8

**Published:** 2019-04-18

**Authors:** Alok Sharma, Artem Lysenko, Yosvany López, Abdollah Dehzangi, Ronesh Sharma, Hamendra Reddy, Abdul Sattar, Tatsuhiko Tsunoda

**Affiliations:** 1 Institute for Integrated and Intelligent Systems, Griffith University, Q, Brisbane, LD-4111 Australia; 2 Laboratory for Medical Science Mathematics, RIKEN Center for Integrative Medical Sciences, Yokohama, Kanagawa Japan; 3 School of Engineering and Physics, Faculty of Science, Technology and Environment, University of the South Pacific, Suva, Fiji Islands; 4 Genesis Institute of Genetic Research, Genesis Healthcare Co, Tokyo, Japan; 5 Department of Computer Science, Morgan State University, Baltimore, MD USA; 6 School of Electrical and Electronics Engineering, Fiji National University, Suva, Fiji; 7 Department of Medical Science Mathematics, Medical Research Institute, Tokyo Medical and Dental University, Tokyo, Japan; 8 CREST, JST, Tokyo, 113-8510 Japan

## Abstract

**Background:**

Post-translational modifications are viewed as an important mechanism for controlling protein function and are believed to be involved in multiple important diseases. However, their profiling using laboratory-based techniques remain challenging. Therefore, making the development of accurate computational methods to predict post-translational modifications is particularly important for making progress in this area of research.

**Results:**

This work explores the use of four half-sphere exposure-based features for computational prediction of sumoylation sites. Unlike most of the previously proposed approaches, which focused on patterns of amino acid co-occurrence, we were able to demonstrate that protein structural based features could be sufficiently informative to achieve good predictive performance. The evaluation of our method has demonstrated high sensitivity (0.9), accuracy (0.89) and Matthew’s correlation coefficient (0.78–0.79). We have compared these results to the recently released pSumo-CD method and were able to demonstrate better performance of our method on the same evaluation dataset.

**Conclusions:**

The proposed predictor HseSUMO uses half-sphere exposures of amino acids to predict sumoylation sites. It has shown promising results on a benchmark dataset when compared with the state-of-the-art method.

The extracted data of this study can be accessed at https://github.com/YosvanyLopez/HseSUMO.

**Electronic supplementary material:**

The online version of this article (10.1186/s12864-018-5206-8) contains supplementary material, which is available to authorized users.

## Background

Post-translational modifications (PTMs) of proteins are enzyme-medicated covalent alterations of protein sequence during which a chemical group can be added to a particular residue or sequence is cleaved at a specific location [1]. These modifications greatly expand the range of possible final forms of proteins that can be generated from the same genomic sequence [2]. PTMs also play an important role in modulation of all aspects of protein function, in particular, they can determine protein localization within the cell [3], mediate signal transduction [4, 5], activate or deactivate enzymes and transporters [6, 7] and underlie protein degradation and recycling [8]. Despite this critical role PTMs play in all living systems, accurate identification of all types of these modifications using laboratory methods remains challenging. Some pertinent problems include [9]: (1) isolation of specific proteins with modification (s) of interest from the highly diverse and biochemically heterogeneous proteome (2) masking effects of the highly abundant proteins in the sample, which can make isolation using standard immunoprecipitation and chromatographic methods difficult and (3) the diversity and molecular complexity of possible PTMs themselves.

Positions at which PTMs are possible are usually highly specific to particular protein residues and are governed by the amino acid motifs at the site and three-dimensional conformation of the protein [1]. Therefore, a priori identification of the sites and proteins where particular PTMs can occur can be an important way to narrow down the possibilities and facilitate their experimental verification. This sequence-based specificity allows for the possibility of identification of these sites using computational predictive approaches and development of such methods is an area of active research [10].

One of the important types of PTMs, called sumoylation, involves reversible covalent bonding of small ubiquitin-like modifier proteins SUMO1, SUMO2 and SUMO3 [11]. This modification is of particular interest due to its involvement in neurodegenerative [12–14] and immune-related diseases [15–17], as well as cancer [18, 19]. Sumoylation involves several enzymatic steps and was previously reported to be commonly found at ψK.E/D (or inverse E/D.Kψ) motif, where ψ can any of the hydrophobic amino acids [20]. However most recent results indicate that as many as half of all sumoylation sites do not actually follow this pattern [4], therefore simple motif-based strategies are likely to be insufficient for good predictive performance on these more recent data.

To date, multiple sequence-based approaches to prediction of sumoylation sites have been proposed. Some earlier approaches are SUMOsp [21], which employs a group-based prediction system (GPS) similarity clustering and SUMOpre [22], which works by fitting a multiple linear regression model to a 7-residue sequence window. In 2009, an updated version of the SUMOsp algorithm was released that achieved higher specificity and accuracy than any other tools available at the time and proved the utility of their original GPS-based approach. Next, SUMOhydro [23] was proposed that introduced an improved dataset of experimentally-determined sumoylation sites and explored an original type of predictive feature (binary-encoded hydrophobicity pattern) in combination with a support vector machine classifier. GPS-SUMO [24] further refined a GPS-based approach proposed in SUMOsp 1.0 and 2.0, and combined it with particle swarm optimization algorithm. Then, an important shift in direction was proposed by [25], who were the first to use protein structural features for prediction of sumoylation sites, in particular, predicted disorder and confirmation flexibility. And, finally, the most recent methods are pSumo-CD [26] and SUMO_LDA [27]. The former applied a covariance discriminant algorithm in combination with pseudo amino acid composition model. The latter works by computing a position-specific amino acid propensity matrix at locations of interest, which was then used in combination with linear discriminant analysis.

Although good performance results have been consistently reported even in the very early studies, it appears likely that these efforts have been severely limited by the amount of experimental data that was available at the time. Most importantly, it appears that the proportion of the sumoylation sites that do not follow the consensus motif was previously greatly underestimated (e.g. it was assumed to be only about 23% in [21]) until sufficient data were collected in more recent studies [4]. This indicates that the problem is both more complex than was previously thought and that structural patterns of the protein fold likely play an important role in determining suitable sumoylation sites. However, a vast majority of previously proposed methods have focused on features chiefly based on amino acid occurrence patterns at the sites of interest. To explore the usefulness of structural features [28–33], we propose a novel method, HseSUMO that uses a combination of four different half-sphere exposure (HSE) measures, originally developed to characterize solvent exposure at particular amino acid residues [34]. We demonstrate that a combination of these features is highly promising for prediction of sumoylation sites and we were able to achieve very good levels of performance even using a relatively simple decision tree classifier (0.89 area under ROC curve for 6, 8 and 10-fold cross-validation schemes).

HSE measure is used for feature computation. It is an alternative measure of the solvent exposure of the amino acids and its use for sumoylation site prediction has shown promising results when compared with the existing state-of-the-art sumoylation site predictor. This indicates that HSE contains the complementary information of the amino acids to identity the sumoylation sites. In the literature, the HSE measure has been shown to contain important information in the related field of research [35, 36].

In addition, the imbalance of data deteriorates the performance. To tackle this, we have used the under-sampling technique to obtain the balanced sampling. Furthermore, to report the statistical significance of the HseSUMO, we performed k-fold cross-validation and reported performance measures such as sensitivity, specificity, accuracy and MCC. For sumoylation site prediction, it is very crucial to have high sensitivity as detecting affected lysine sites are of prime importance. Thus, HseSUMO achieved performance improvement of 36.5% in terms of sensitivity and 16.7% in terms of accuracy.

## Methods

### Dataset description

All data used in this analysis is from the Compendium of Protein Lysine Modifications (CPLM) database [37], a resource which manually curates information about 12 different types of lysine PTMs from literature. From these data we have identified a subset of all proteins which were profiled for sumoylation, giving 448 proteins in total that had 780 positive examples of sumoylated sites and 21,353 confirmed non-sumoylated sites. We filtered out sequences over 40% sequential similarity using CD-HIT [38]. Therefore, our benchmark dataset has less than 40% sequential similarity. The difference between positive and negative samples creates class imbalance problem. The two commonly used strategies to overcome this problem are over-sampling and under-sampling. The over-sampling procedure could increase the probability of over-fitting the model, and under-sampling often provides a modest solution for a given model. Therefore, we selected under-sampling procedure like NearMiss method [39] by employing the imbalanced-learn package of python. After applying NearMiss method we ended up with 780 negative and 780 positive samples.

### Half-sphere exposure feature computation

Half-sphere exposure (HSE) is a solvent exposure measurement similar to Contact Number (CN) [40], and Accessible Surface Area (ASA) [41]. In contrast to ASA and CN that do not provide explicit information regarding the orientation of side chains which is important on the conformation of the 3D structure of the proteins and its interaction with other macro-molecules HSE is designed to attain such information [42]. HSE is introduced in [42] and can be calculated in two ways by splitting the sphere around the *C*α atom (with radius R typically equal to 12 Å) into two half-spheres either along the vector of *C*α-*C*β atoms or a pseudovector of *C*α-*C*β generated from the sum of vectors *Cα*_*i* − 1_ − *Cα*_*i*_ and *Cα*_*i* + 1_ − *Cα*_*i*_. The first one is referred to as HSEβ and the second one as HSEα.

**Fig. 1:**
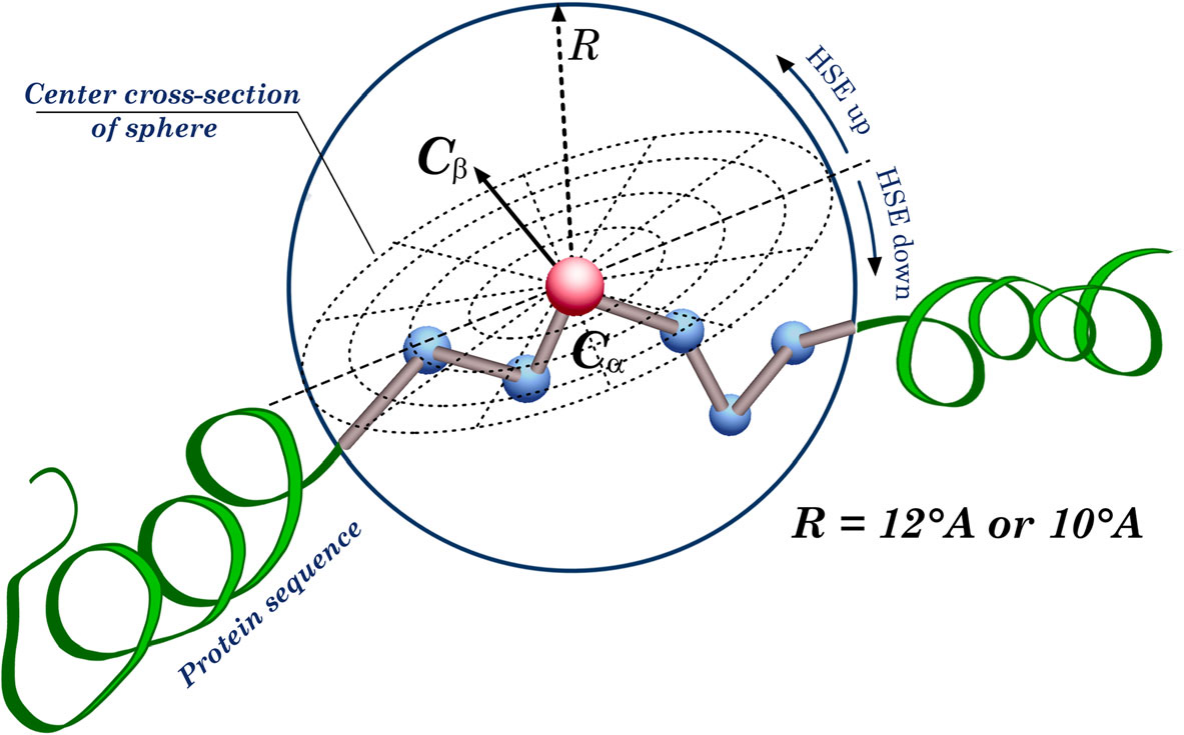
An illustration of half-sphere exposure of amino acid

For HSEβ, the half-sphere containing the *C*β atom is then defined as upper and the other as down half-spheres and the numbers of *C*α atoms enclosed in these two half-spheres were named as HSEβ-up and HSEβ-down, respectively. An illustration for HSE is given in Fig. 1. For HSEα, the half-sphere perpendicular to the sum of *Cα*_*i* − 1_ − *Cα*_*i*_ and *Cα*_*i* + 1_ − *Cα*_*i*_ vectors is HSEα-up and another one is HSEα-down. The main difference between HSEβ and HSEα is that calculating the second does not require the position of *C*β which is hard to determine for some cases. It was shown in [42] that HSEα is a better measurement for solvent exposure than CN, ASA and even HSEβ. However, the use of HSEα and HSEβ simultaneously has shown to be complementary [43, 44].

### Lysine residue description

Lysine residue (sumoylated or non-sumoylated) is described by a segment of 31 amino acids (15 upstream and 15 downstream) as done by previous studies [45–51]. If a lysine residue is present in any terminus of a protein sequence and if the segment of 31 residues is not possible then we adjusted the segment by employing mirror of amino acids [45]. Suppose lysine residue site is denoted by *K* then a segment of 31 amino acids will be given as *S* = {*A*_−15_, *A*_−14_, …, *A*_−1_, *L*, *A*_1_, …, *A*_14_, *A*_15_}. Therefore, a lysine *K* consists of 31 HSE features and it is characterized by the 124-dimensional feature vector. The high dimensionality can be further reduced by feature selection techniques [52–54].

### Model training

The model was trained using a decision tree-based classifier. Despite its simplicity, the decision tree classifier has the advantage of allowing easy interpretation of the underlying model, which can facilitate discovery of biologically meaningful patterns captured by the model. To get an accurate measure of performance we have used a repeated cross-validation approach. There are three cross-validation methods, i.e., independent dataset test, sub-sampling (or K-fold cross-validation) test, and jackknife test, often used to evaluate the anticipated success rate of a predictor. Among the three methods, however, the jackknife test is deemed the least arbitrary and most objective one and hence has been widely recognized and increasingly adopted by investigators to examine the quality of various predictors [55–59]. In the jackknife test, each sequence in the training dataset is in turn singled out as an independent test sample and all the rule-parameters are calculated without including the one being identified. Though Jackknife test is the most effective, due to its computational expense we have adopted the K-fold test which is an alternative evaluation method and has been widely used in many protein-related problems [33, 60–62]. Briefly, in K-fold the dataset was partitioned into *k* approximately equally-sized folds (in this case either 6, 8 or 10) [63]. In turn, each of the subsets was set aside and used for validation of the model trained on the remaining *k-1* folds. To train the predictor, we used the Python implementation of decision trees. The quality of a split was measured with Gini impurity and “best” was selected as the strategy for choosing the split at each node. Moreover, only two samples were required for splitting an internal node.

### Evaluation of model performance

According to established evaluation framework, we define the number of instances where sumoylated sites were predicted, either correctly or incorrectly as true positives (TP) or false positives (FP) respectively. Similarly, for non-sumoylated sites, counts of correct or incorrect predictions are defined as false negatives (FN) or true negatives (TN). Then sensitivity (also known as true positive rate) is a proportion of correctly predicted sumoylated sites among all real sites:

1 $$ Sens=\frac{TP}{FN+ TP} $$

and specificity is a proportion of all correctly predicted non-sumoylated sites among all negative predictions:

2 $$ Spec=\frac{TN}{FP+ TN} $$

The other two performance metrics are accuracy and Matthew’s correlation coefficient which are defined according to the following formulae:

3 $$ Accuracy=\frac{TN+ TP}{TN+ FN+ TP+ FP} $$

4 $$ MCC=\frac{\left( TP\times TN\right)-\left( FP\times FN\right)}{\sqrt{\left( TN+ FP\right)\times \left( TP+ FN\right)\times \left( TP+ FP\right)\times \left( TN+ FN\right)}} $$

The last evaluation metric used was the area under receiver-operator characteristic (ROC) curve. The curve is computed by considering how the trade-off between sensitivity and false positive rate changes at a range of different cut-offs of class prediction probability (*M*) returned by a given classifier. Here, the false positive rate is defined as follows:

5 $$ FPR=\frac{FP}{TN+ FP} $$

And, finally, given these definitions the area under (AUC) the ROC curve is described as follows:

6 $$ AUC(M)={\int}_{\infty}^{-\infty } Sens(M)\times \left(-{FPR}^{\prime }(M)\right)\  dM $$

**Fig. 2:**
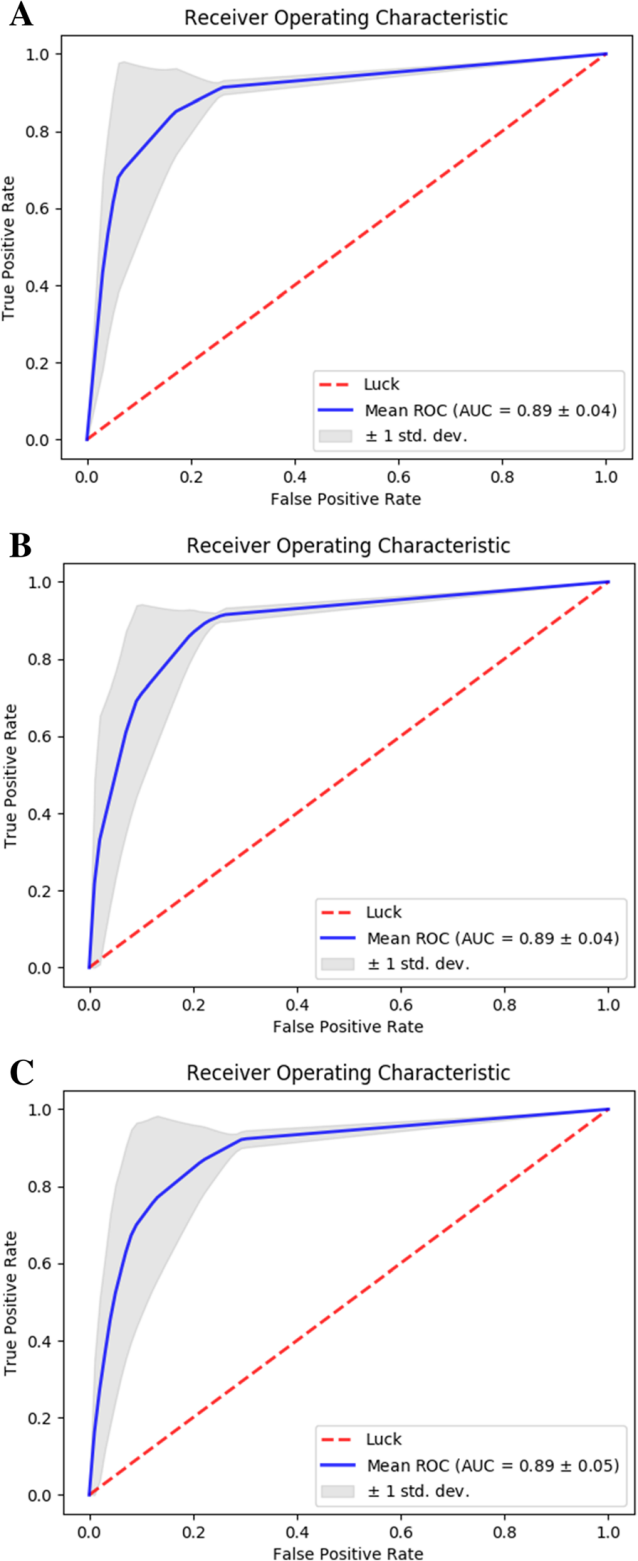
Receiver operating characteristic curves of HseSUMO performance. Three panels show results for 6-fold (**a**), 8-fold (**b**), and 10-fold (**c**) evaluation schemes

To verify that the performance of the classifier is robust, ROC-AUC measures were averaged across all cross-validation folds with the same fold number, and the resulting average curves, AUC values and their standard deviations are shown in Fig. 2. We also report the performance of the most recently released alternative method called pSumo-CD. The comparison was done by annotating all proteins in our dataset using the pSumo-CD web server. The resulting annotations were processed in an identical way to those of our method and the same set of four performance metrics were computed.

## Results and discussion

The results of our evaluation are shown in Table 1, which showed that our method was able to outperform pSumo-CD on according to all metrics with exception of specificity. However, we note that there is a trade-off between the sensitivity and specificity measures, which can be realized by altering a threshold of the classifier at which a particular prediction is made. Notably, the sensitivity of pSUMO-CD is considerably lower, and more robust measures like accuracy (16.7% higher) and Matthew’s correlation coefficient (0.296 higher) are considerably better for our method. The difference between performances achieved for different *k*-folds validation schemes was also relatively small, indicating that over-fitting is likely to be relatively low. We also would like to point out that as pSumo-CD is, in essence, a machine learning method it will inherently have better performance on the samples from the original dataset used to train its model. Although from the description of the method we were able to deduce that there might be an overlap between our evaluation dataset and training dataset of pSumo-CD, not enough information was provided to allow us to identify and exclude these samples. Therefore, for this reason, the evaluation is likely to be biased in favor of pSumo-CD.

**Table 1:** Performance evaluation of HseSUMO. CV refers to the cross-validation scheme for 6-fold, 8-fold and 10-fold

Methods	Sensitivity	Specificity	Accuracy	MCC
pSumo-CD	0.536	**0.921**	0.728	0.494
HseSUMO (CV- 6	0.895	0.895	**0.895**	**0.790**
HseSUMO (CV- 8)	0.897	0.879	0.888	0.777
HseSUMO (CV- 10)	**0.904**	0.872	0.888	0.776

The highest values are depicted as bold faces

To compare the HseSUMO with pSumo-CD predictors, we have analyzed the sumoylation sites predicted by HseSUMO and pSumo-CD. (Additional file 1: Supplementary Table S1) shows the total number of sites predicted for 448 proteins analyzed in this paper. It is observed that out of 780 positives sites, HseSUMO correctly predicted 698 sites compared to 418 sites predicted by pSumo-CD. Thus, 35% increase in the prediction ratio is noted for HseSUMO. Moreover, evaluating the performance of HseSUMO for different folds resulted with higher value of sensitivity for 10-fold cross-validation, while for 6-fold cross-validation a better performance was observed in terms of specificity, accuracy and MCC measures. Overall, there is a very little change in the performance measures for different folds when comparing HseSUMO and pSumo-CD.

In detail, the highest sensitivity by HseSUMO was noted as 0.904. The specificity was in the range of 0.872 and 0.895. The specificity of pSumo-CD was the highest achieving at 0.921, however, it provided sensitivity of 0.536 only. Thus, most of the sumoylation sites were not detected. The accuracy for HseSUMO was in the range of 0.888 and 0.895, however, for pSUMO-CD we achieved 0.728. The MCC measure was in the range 0.776 and 0.790 for HseSUMO, while pSUMO-CD was able to achieve 0.494 MCC score. In summary, we were able to achieve performance improvement of 36.5, 16.7, and 29.6% in terms of sensitivity, accuracy and MCC, respectively. However, pSumo-CD only showed high performance on specificity measure.

Although the metrics reported above are commonly used to evaluate the performance of particular machine learning classifiers, they are all subject to a limitation of only being applicable after a class prediction has been made. However, most classifiers can return a score rather than hard prediction and the final prediction is generated by applying a particular cut-off to that score. Depending on the cost attached to making an error of particular type different threshold can be chosen. Therefore, an analysis of the area under the ROC curve, which summarizes performance at all possible threshold can potentially be more useful. The results of the ROC-AUC analysis of our method are shown in Fig. 2; average AUC value for all fold numbers was recorded at 0.89, indicating stable performance. In all cases, the higher standard deviation was associated with lower score cut-offs.

Our method was able to achieve highly competitive results despite forgoing pattern-based features that were key for many previous approaches, and, importantly, also performed well on an updated dataset that incorporated greater proportion of sites. These results indicate that structural features, e.g. HSE features are likely important for underlying biology of sumoylation mechanism and could be highly promising features for improvements in computational prediction of both sumoylation sites as well as other types of PTMs.

## Conclusions

Despite recent progress in the development of better laboratory-based PTM detection methods, their experimental identification remains challenging. In this study, we propose a new, accurate method for prediction of sumoylation sites, an important type of PTM underlying multiple human diseases. Our method demonstrates the predictive power of features based on protein structure in case of sumoylation. This finding is of great interest, as all of the currently available methods in this area are based on features derived from some form of amino acid co-occurrence patterns, but recent experimental results indicate that over half of all known sumoylation sites are not associated with a clear amino acid motif [4]. Furthermore, to make current predictors more practical to the scientific community, a user friendly web-server is often developed [36, 64–66]. Therefore, we will make an effort to provide a flexible web-server for the method in the near future, which will undoubtedly contribute to enhance the ongoing work of experiment scientists and medical researchers alike. Meanwhile, interested researchers can access to the scripts and training matrices available at https://github.com/YosvanyLopez/HseSUMO.

## Additional file

Additional file 1: Supplementary **Table S1.** Prediction of protein sequences by predictors (XLS 58 kb)

## Abbreviations

ASA: Accessible surface area
AUC: Are under curve
CN: Contact number
FN: False negative
FP: False positive
HSE: Half-sphere exposure
HseSUMO: Sumoylation predictor using HSE of amino acids
MCC: Matthew’s correlation coefficient
PTM: Post-translation modification
ROC: Receiver operating characteristics
TN: True negative
TP: True positive
